# Perivascular spaces around arteries exceed perivenous spaces in the mouse brain

**DOI:** 10.1038/s41598-024-67885-y

**Published:** 2024-07-26

**Authors:** Nina G. Smets, Shakira A. van der Panne, Gustav J. Strijkers, Erik N. T. P. Bakker

**Affiliations:** 1 Department of Biomedical Engineering and Physics, Amsterdam University Medical Center, Amsterdam, The Netherlands; 2 Amsterdam Neuroscience Research Institute, Amsterdam, Netherlands; 3 Amsterdam Cardiovascular Sciences Research Institute, Amsterdam, Netherlands

**Keywords:** Cerebrospinal fluid, Perivascular space, Veins, Glymphatics, Meninges, In vivo two-photon imaging, Diseases of the nervous system, Neuro-vascular interactions

## Abstract

The perivascular space (PVS) surrounds cerebral blood vessels and plays an important role in clearing waste products from the brain. Their anatomy and function have been described for arteries, but PVS around veins remain poorly characterized. Using in vivo 2-photon imaging in mice, we determined the size of the PVS around arteries and veins, and their connection with the subarachnoid space. After infusion of 70 kD FITC-dextran into the cerebrospinal fluid via the cisterna magna, labeled PVS were evident around arteries, but veins showed less frequent labeling of the PVS. The size of the PVS correlated with blood vessel size for both pial arteries and veins, but not for penetrating vessels. The PVS around pial arteries and veins was separated from the subarachnoid space by a thin meningeal layer, which did not form a barrier for the tracer. In vivo, FITC-dextran signal was observed adjacent to the vessel wall, but minimally within the wall itself. Post-mortem, there was a significant shift in the tracer's location within the arterial wall, extending into the smooth muscle layer. Taken together, these findings suggest that the PVS around veins has a limited role in the exchange of solutes between CSF and brain parenchyma.

## Introduction

Neurodegenerative diseases, including Alzheimer’s disease (AD) and cerebral amyloid angiopathy (CAA) are characterized by the accumulation of toxic protein aggregates. Accumulation of proteins such as amyloid-β around blood vessels and in the brain tissue has led to the suggestion that dysfunctional brain clearance is a common denominator in several types of dementia^1^. Clearance of waste proteins occurs through local degradation, extrusion across the blood–brain barrier, and via the perivascular space (PVS)^2^. Several theories exist regarding brain clearance via the PVS, including the glymphatic model, the intramural periarterial drainage (IPAD) model, and the mixing hypothesis^3^. These views vary with respect to the anatomical location of the perivascular drainage route, i.e. either along basement membranes within the arterial wall, or directly adjacent to the vessel wall. Furthermore, there are differing opinions on the direction of solute transport and the forces that drive solute clearance. For review of these hypotheses see^3–5^.

Notwithstanding the different views on brain clearance routes, the PVS is generally believed to be involved in waste removal from the brain. Iliff et al. introduced the term “glymphatic system” for the perivascular clearance of waste removal, proposed a pathway where tracers injected into the CSF enter perivascularly along arteries, then move into the interstitium via the PVS along penetrating arteries, mix with interstitial fluid, and finally exit via the PVS along veins^6^. In this seminal paper, Iliff et al. showed through post-mortem analysis of tissue sections that fluorescent tracer signal around the venous PVS was detected at a later stage^6^. This finding led to the conclusion that this pathway served as the exit route for waste. Since then, most research focused on the PVS around arteries. We and others reported pulsatile flow in the PVS along arteries, which could serve as one of the driving forces for transport^7,8^. The size of the PVS can be quantified in relationship to blood vessel size. PVS-to-vessel area ratios of pial arteries have been analyzed before, but the PVS around veins and penetrating arteries has been disregarded or not segmented^9,10^. Recent studies have provided further detailed descriptions of the arterial PVS^11^. However, the PVS around veins has received significantly less attention, and there is a notable lack of in vivo quantitative data concerning venous PVS.

In this study we aimed to investigate several essential characteristics of the PVS around veins, including size, shape, and abundance, which are essential to understand the brain’s waste removal pathways, but are yet to be fully understood. We anticipate that PVS around veins are smaller than PVS around arteries, based on the finding that the vast majority of PVS detected by MRI are associated with arteries^12^. Therefore, we judged optical imaging methods to be better suited for this purpose. Thus, we used in vivo two-photon microscopy and post mortem histology in mice, to characterize both the arterial and the venous PVS along pial and penetrating vessels. Moreover, we investigated possible interconnections of the PVS between arteries and veins at the level of the subarachnoid space, considering possible clearance routes. Lastly, we studied the location of the PVS with respect to the vessel wall itself, given that both intramural and extramural routes have been identified^4^.

## Materials and methods

### Animals

In this study, 15 male and female C57BL/6J mice of 16 weeks of age were examined (n = 8 males, and n = 7 females). Animals were obtained from Envigo RMS (The Netherlands), and were acclimatized for at least one week before entering the study. All experimental studies were approved by the animal ethics committee of the University of Amsterdam, and were executed according to the European Union guidelines for the welfare of laboratory animals (Directive 2010/63/EU). Animals were housed in groups with a 12-h light/dark cycle and ad libitum access to food and water. All experimental procedures were carried out at the same experimental day after intraperitoneal injection of anesthesics using an induction mixture of 125 mg/kg ketamine (Ketanest-S, Pfizer), 0.2 mg/kg dexmedetomidine (Dormitor; Orion Pharma) diluted in NaCl (0.9%) and applied as a volume with a dose of 0.0075 ml/gram bodyweight. Maintenance dose was administered intraperitoneal using 1/3th of the normal dose.

### Cranial window surgery

Animals were anesthetized, fixed in a stereotactic frame, and positioned on a heating pad. A temperature probe was rectally inserted to monitor and maintain body temperature. Eyes were kept hydrated using an ocular lubricant (Duratears, Alcon). During the entire experimental procedure, 100% oxygen was administered via a nose cone. The skin was removed from the upper part of the skull and an additional local anesthetic (1% xylocaine) was applied onto the skull before removal of the periosteum. A circle of 3 mm diameter was precisely crafted above the right middle cerebral artery (MCA) using a dental drill with round tip. Throughout the drilling process, the skull was constantly cooled with PBS to prevent damage from overheating. Next, the circular skull section was removed carefully leaving the dura intact. This offered a clear view on the cerebral vasculature. The window was sealed using a small circular glass cover glass and a metal ring was glued onto the skull for fixation of the animal under the microscope.

### Tracer injection

After skin incision, neck muscles were parted in order to visualize the cisterna magna. FITC-dextran 70 kDa (5 mg/ml, Invitrogen, fixable, D1822) was diluted in artificial CSF (148 mM NaCl, 3 mM KCl, 1.4 mM CaCl_2_·2H_2_O, 0.8 mM MgCl_2_·6H_2_O, 0.8 mM Na_2_HPO_4_·7H_2_O, 0.1 mM NaH_2_PO_4_·7H_2_O). 15 µl of the mixture was injected with a 30-gauge needle into the CSF through the cisterna magna at a rate of 1 µl/minute using a pump (Harvard Apparatus). The needle was secured with tissue glue afterward to prevent movement. Texas Red-dextran 70 kDa (10 mg/ml, Invitrogen, fixable, D1864) was diluted in PBS and 150 µl of the solution was administered into the bloodstream via retro-orbital injection.

### Two-photon microscopy

We employed a Leica multiphoton microscopy system (Leica Microsystems, Wetzlar, Germany) with an adjustable Insight DeepSee (SpectraPhysics) laser for imaging the brain's surface. Initially, we used a 2.5 × dry objective paired with a CCD camera through light microscopy for orientation, then switched to a 25 × water immersion objective (NA = 0.95) for two-photon microscopy. An excitation wavelength of 790 nm was used for FITC-dextran, and 910 nm for Texas Red-dextran. Emitted light was captured by two HyD detectors, using a filter cube for FITC-TRITC signal with a beam splitter at 560 nm. The fluorescence measured at 525 nm with bandwidth of 50 nm was labeled as FITC, while the signal measured at 585 nm with bandwidth of 40 nm was labeled as Texas Red fluorescence. Laser power was approximately 55 mW for observations at the surface and increased accordingly for observations at increasing depths within the parenchyma. Images were acquired with a matrix of 1024 × 1024 pixels. Z stacks were made reaching to a depth of approximately 200 µm into the tissue for both arteries and veins. Images were taken from 15 min until one hour after the finalization of the CSF injection. The time point was chosen as a time point where tracers are expected to label both entry and exit routes. We do not think that the dimensions of PVS or connections with the SAS would be different at other time points. However, we do acknowledge that the amount of tracer associated with arteries vs veins could be different at other time points. Images were processed with Fiji ImageJ software^13^. Image stacks were reformatted in slices at 90-degrees angle to vessel wall for PVS and vessel size measurements. Perivascular spaces were delineated manually. Arteries were distinguished from veins based on the presence of arterial pulsations and morphology, which included wall thickness and arteries crossing over veins in the SAS (Supplementary Video 1). The PVS of penetrating arteries and ascending veins was measured at the point of entry and exit from the brain tissue. Each bifurcation of a blood vessel into two daughter branches was considered a separate segment and included in the measurements. For the quantification of the signal intensities, we normalized the signal intensity to that of the background in the image.

### Perfusion fixation

Following imaging, after approximately one hour of tracer infusion, the mouse received an intraperitoneal injection of 0.1 ml heparin and was subsequently sacrificed using an overdose of anesthesia (3 times the induction dose). The thorax was opened, a needle was inserted into the left ventricle and the right atrium was punctured. The mouse was perfused with PBS to drain the blood after which perfusion was continued with 15 mL of 4% paraformaldehyde (PFA) for tissue fixation. Then, the brain was extracted for further processing. Brain tissue was fixed for 24 h in PFA at 4 °C. Next, brain tissue was cryoprotected in 30% sucrose solution for maximum 48 h at 4 °C. The midbrain was separated and stored at − 80 °C.

### Immunofluorescence staining

For a subset of six animals, including two females and four males, we performed additional immunohistochemistry and light-sheet microscopy of stained brain tissue sections. Coronal brain sections of 80 µm thickness were cut using a cryomicrotome (Thermo Cryostar NX70 Cryostat). Our analysis primarily centered on sections extracted approximately 1.28 mm anterior to the Bregma. Sections were stored in a cryoprotectant solution (28% sucrose and 30% ethylene glycol in PBS) at -20 °C until further use. The free-floating sections were stabilized for 30 min. at room temperature and washed with PBS followed by incubation with blocking solution (3% BSA, 2% Tween 20, and 5% donkey serum in PBS) for 1h at room temperature. Next, sections were incubated overnight at room temperature with primary antibody rabbit anti-mouse laminin (1:250; Sigma-Aldrich, #L9393) diluted in blocking solution. On the next day, sections were washed with PBS and incubated overnight at room temperature with secondary antibody goat anti-rabbit Cy3 (1:200; Jackson ImmunoResearch, #111-165-144) diluted in 1% BSA in PBS. The sections were washed with PBS and mounted on Superfrost microscopy slides (Thermo Fisher Scientific). The slides were sealed using Vectashield with DAPI (Vector Labs) and a cover glass, and left to dry for 20 min. at room temperature followed by storage at 4°C for at least 24 h before imaging.

### Light-sheet microscopy and image analysis

Images were captured using a Leica TCS SP8 DLS microscope (Leica Microsystems, Wetzlar, Germany). Per animal, one section with the least cutting damage was selected for imaging. For each section, a z-stack (2 µm steps) tile scan (512 × 512 matrix per tile) was made of the cortex of one hemisphere using a 40 × oil objective. The laser power and gain were adjusted per section for over- and underexposure. For each hemisphere imaged, we counted the total number of surface and penetrating veins and arteries exhibiting FITC fluorescence. Visual inspection of the vessels in the z-stack was conducted in order to distinguish veins from arteries. Objective criteria for this assignment included the shape and branching of the vessels, visibility of smooth muscle cells and elastin, and vessel wall thickness. Vessels with no clear arterial or venous structure were excluded from further analysis.

For each sample, the z-stack of the FITC-dextran channel was converted into a maximum intensity projection which was then loaded into ImageJ Fiji software (https://imagej.net/software/fiji/) for analysis of tracer quantity along the penetrating vessels. A region of interest was placed around the vessel to calculate the total arterial or venous signal (integrated density). The background signal was measured by placing the same region of interest next to the vessel. A background-corrected arterial or venous signal was measured by subtracting the background signal from the total arterial or venous signal. An average intensity (average integrated density) was calculated for the arteries and veins in each sample, as well as a total intensity (total integrated density).

### Electron microscopic database

Electron microscopic (EM) images were obtained from an open data source^14^. This database contains a stack of EM images representing one cubic millimeter of a mouse visual cortex, which provides a useful reference to identify relevant anatomical structures observed in the in vivo images.

### Statistical analysis

Statistical analyses were performed using Graphpad Prism 9 software. Correlations were analyzed using a linear regression analysis. A non-parametric Mann–Whitney test was done if data was not normally distributed. Data was not normally distributed for the sizes of PVS and blood vessels, and the signal intensities of the vessel wall. Paired t-tests were conducted to study the difference between the ex vivo number of pial and penetrating arteries and veins, the average integrated density of the arteries and veins, and the sum of the integrated densities of the arteries and veins. All values are presented as mean ± standard deviation.

### Ethics approval

All experimental studies were approved by the animal ethics committee of the University of Amsterdam, and were executed according to the European Union guidelines for the welfare of laboratory animals (Directive 2010/63/EU).

## Results

### Morphology of perivascular spaces around arteries and veins

**Figure 1:**
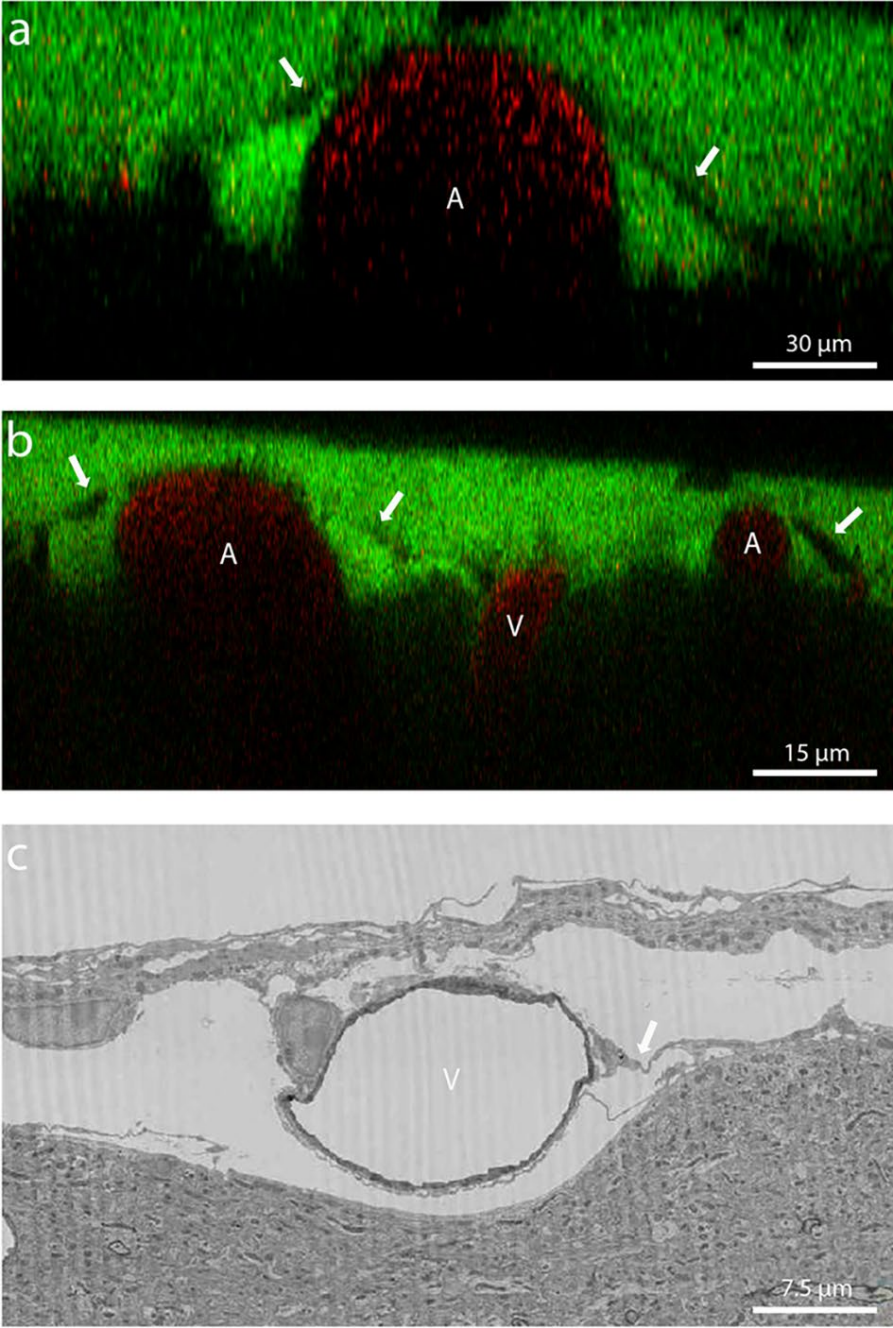
(**a**) In vivo two-photon microscopic image of a perivascular space, separated from the subarachnoid space by a thin membrane (white arrow). (**b**) In vivo two-photon microscopic image of the membrane covering multiple blood vessels in the subarachnoid space. Red = blood vessel; green = CSF tracer, *A* artery, *V* vein. (**c**) Image from the EM database showing a membrane between the perivascular space and the subarachnoid space.

The PVS was visible as two triangular spaces next to both arteries and veins. The PVS was separated from the subarachnoid space by a thin membrane (Fig. 1a,b), which allowed a clear segmentation of the PVS. In order to identify the nature of this membrane in more detail, we consulted the online electron microscopy database wherein one cubic millimeter of mouse brain is searchable^14^. In this database remnants of this specific membrane are found surrounding vessels in the subarachnoid space (Fig. 1c). We therefore think that the membrane gets easily damaged or displaced during postmortem fixation and processing. According to literature, it could be an extension of the pia mater as described by Weller et al.^15^, a structure Pizzo et al. characterized as porous leptomeningeal sheets^16^. Alternatively, it might be the membrane described as SLYM^17^. The membrane not only enveloped individual pial vessels but also extended over multiple blood vessels, particularly when these vessels intersected or were situated near each other.

### Perivascular spaces are larger around arteries as compared to veins

The PVS around both pial arteries and veins as well as around the penetrating arteries and ascending veins were segmented in two-photon microscopy images obtained in vivo (Fig. 1a,b). All pial arteries were accompanied by FITC positive perivascular spaces (n = 24). However, this was only true for 16 out of the 26 pial veins. Similar patterns were observed in the penetrating blood vessels where all arteries demonstrated PVS signal, while only 7 out of 19 penetrating veins were positive for CSF tracer. Pial veins that showed labeling generally were close to arterial PVS, or crossing arteries.

**Figure 2:**
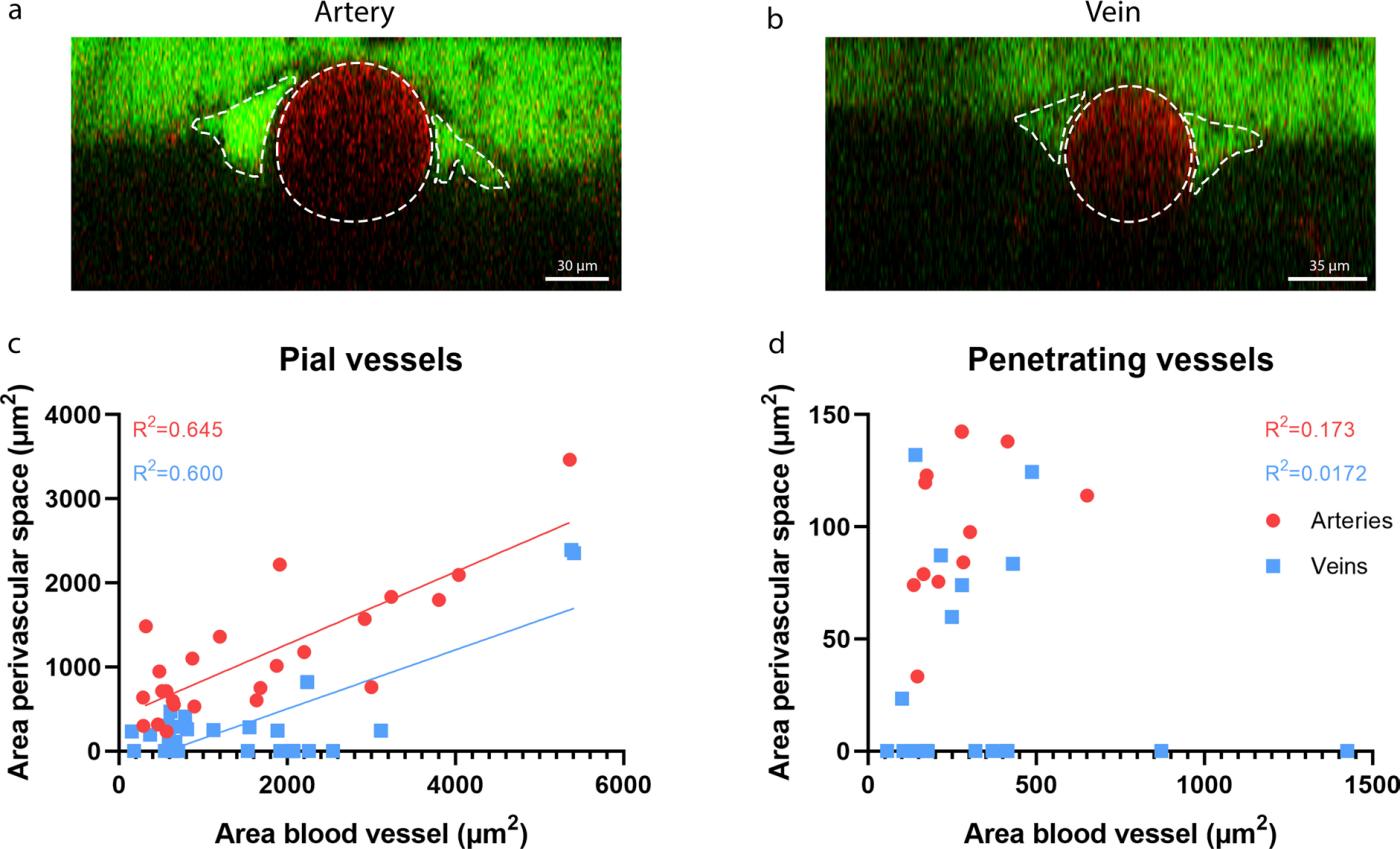
Example of the segmentation of the perivascular space of (**a**) a pial artery, or (**b**) a pial vein. Surface area of the perivascular space (µm^2^) plotted against the cross-sectional area of the blood vessel (µm^2^) in (**c**) pial blood vessels and (**d**) penetrating blood vessels. Data was obtained from 11 mice. Red = blood vessel; green = CSF.

Periarterial spaces were larger compared to perivenous spaces around pial vessels (p < 0.0001). The same was true for penetrating vessels (p = 0.0002). In the SAS, the size of the PVS scaled with blood vessel size, i.e. a larger cross-sectional area of the artery was associated with a larger cross-sectional area of the PVS (R^2^ = 0.645, p < 0.0001, N = 24; Fig. 2c). A similar correlation was seen for the PVS around pial veins in the SAS (R^2^ = 0.5995, p < 0.0001, N = 26). Around penetrating vessels, no significant correlations were observed between vessel and PVS sizes around both arteries and veins in this dataset (arteries: R^2^ = 0.173, p = 0.179, N = 12; veins: R^2^ = 0.0172, p = 0.593, N = 19; Fig. 2d).

### Perivascular spaces of arteries and veins are continuous via the subarachnoid space

**Figure 3:**
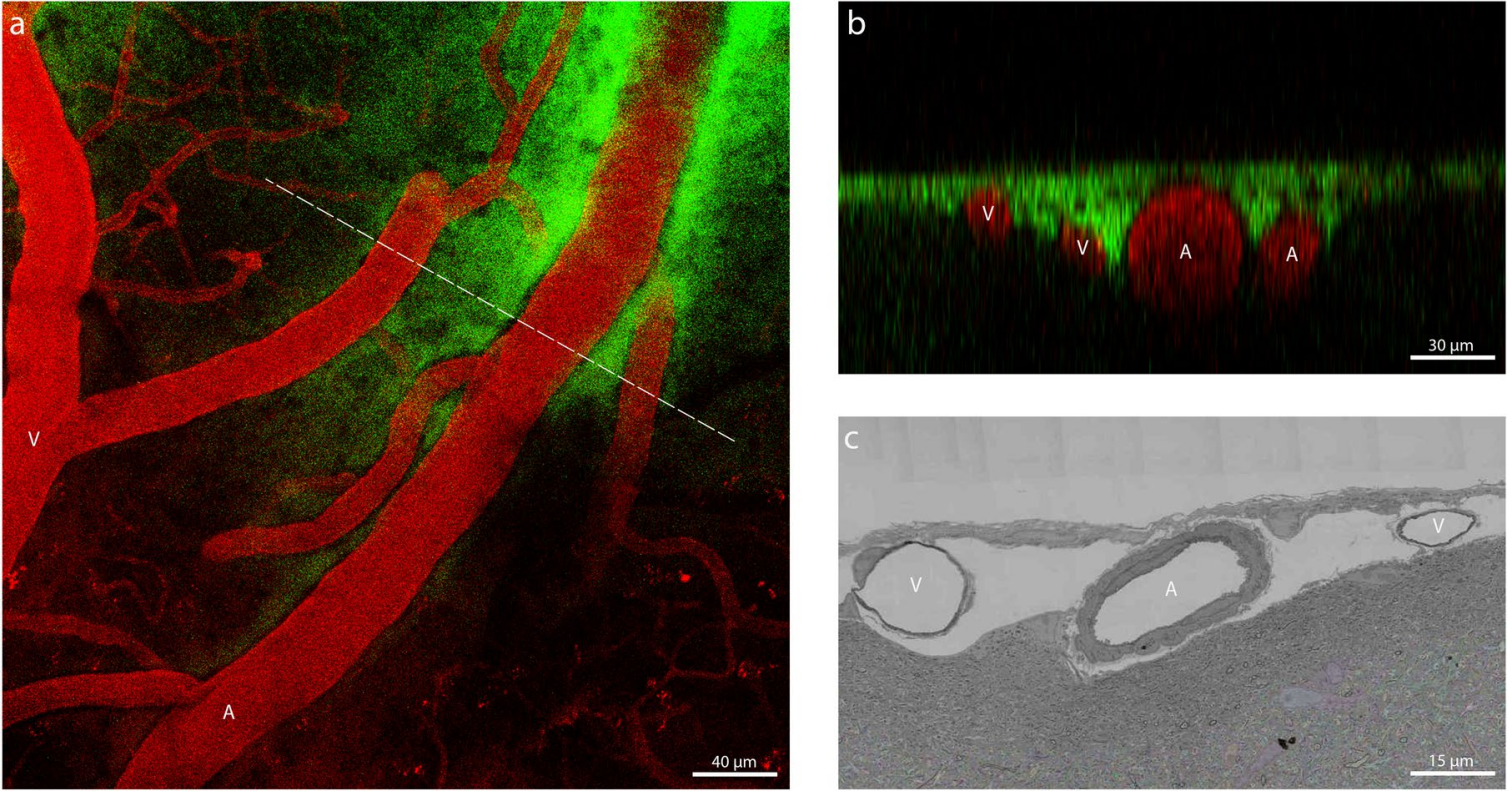
(**a**) Maximum intensity projection of a two-photon imaging stack. A pial artery (A) and vein (V) with side branches are represented in red. The subarachnoid space (SAS) and perivascular spaces are visualized with FITC-dextran (green). (**b**) Cross-sectional image at the location of the dashed line in A. (**c**) EM image taken from www.microns-explorer.org.

While many veins lacked perivascular FITC-dextran signal, a subset exhibited noticeable signal. These were typically at locations were arteries and veins crossed, or where arteries and veins were located in close proximity. Figure 3a shows an example of a pial artery and vein in close proximity, with FITC-dextran present around both vessels and their side branches. The FITC-dextran signal, visible in both longitudinal and cross-sectional views, seamlessly extended between the vessels through the subarachnoid space, despite the presence of the meningeal membranes (Fig. 3b). This closely mirrored the anatomical details seen in the EM images, as highlighted in Fig. 3c.

### Perivascular tracer penetrates the vessel wall post mortem

**Figure 4:**
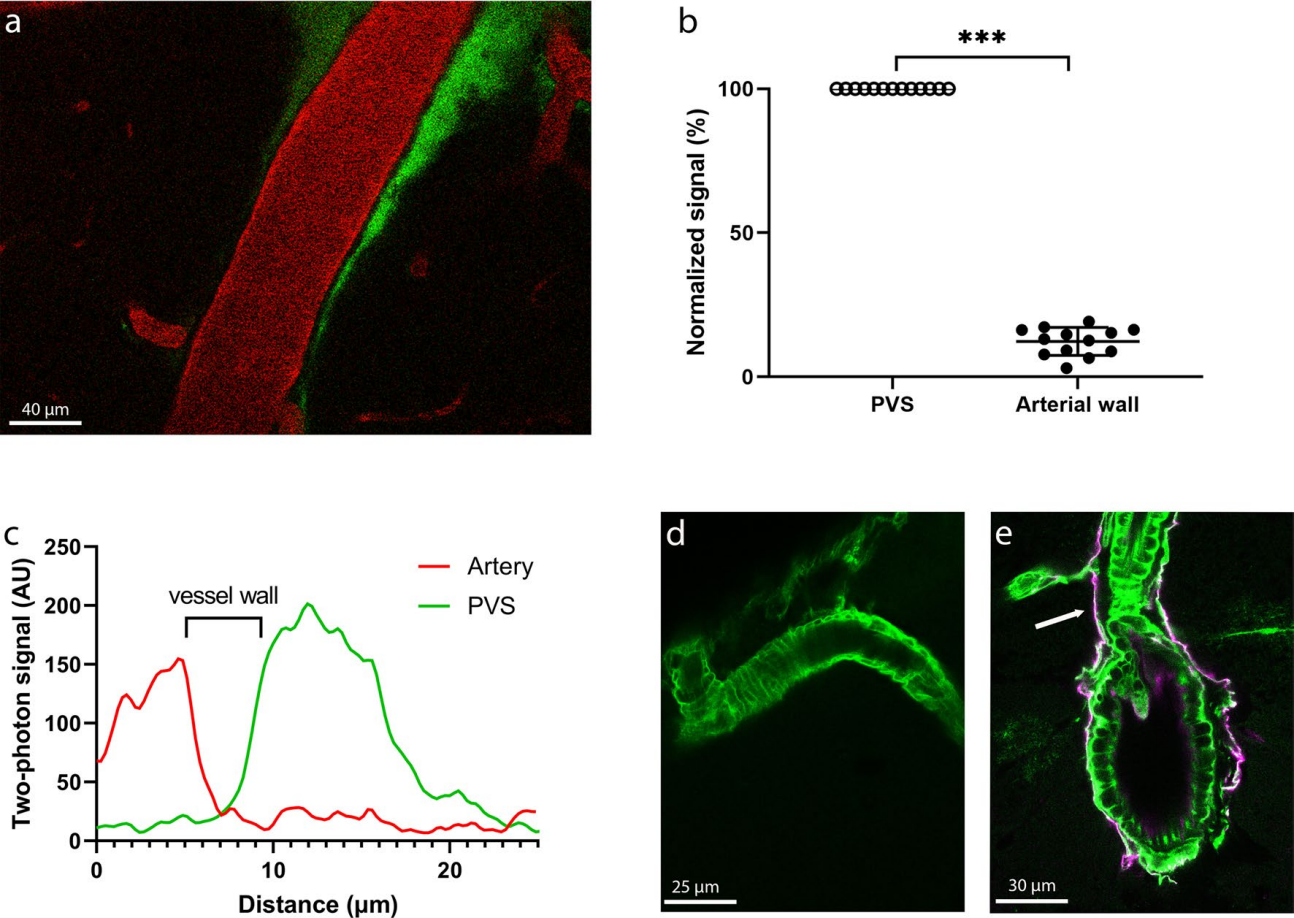
(**a**) Example of in vivo two-photon microscopy image of an artery with a vessel wall apparent as dark structure between the lumen and PVS. (**b**) Normalized signal intensity for the PVS and the arterial wall (n = 13). (**c**) Signal intensity profiles across the artery, vessel wall, and PVS. (**d**) Post mortem example of an artery with FITC PVS signal leaking into the arterial wall. (**e**) Post mortem example of a cross sectioned artery with FITC signal around smooth muscle cells of the arterial wall. Red = blood vessel; green = perivascular space; pink = laminin; arrow = perivascular space.

While the vessel wall of veins was very thin, the arterial wall was clearly visible using in vivo 2-photon microscopy as a dark line, with a minimum amount of tracer (Fig. 4a). Quantification of the signal intensity of the arterial wall confirmed that significantly lower signal was present in the wall as compared to the PVS adjacent to the vessel wall (PVS: 100% vs arterial wall 12.3 ± 4.9%, p < 0.001, N = 13; Fig. 4b). An example of a signal intensity profile for PVS and blood vessel is presented in Fig. 4c.

The distribution of the tracer observed in vivo underwent significant alterations during histological processing of the tissues, as depicted in Figs. 4d,e. Consequently, in post-mortem analyses of the same brain, the perivascular tracer was found to have infiltrated the arterial wall. The PVS itself was now devoid of signal.

### Tracer signal associated with penetrating arteries was larger compared to signal associated with ascending veins

**Figure 5:**
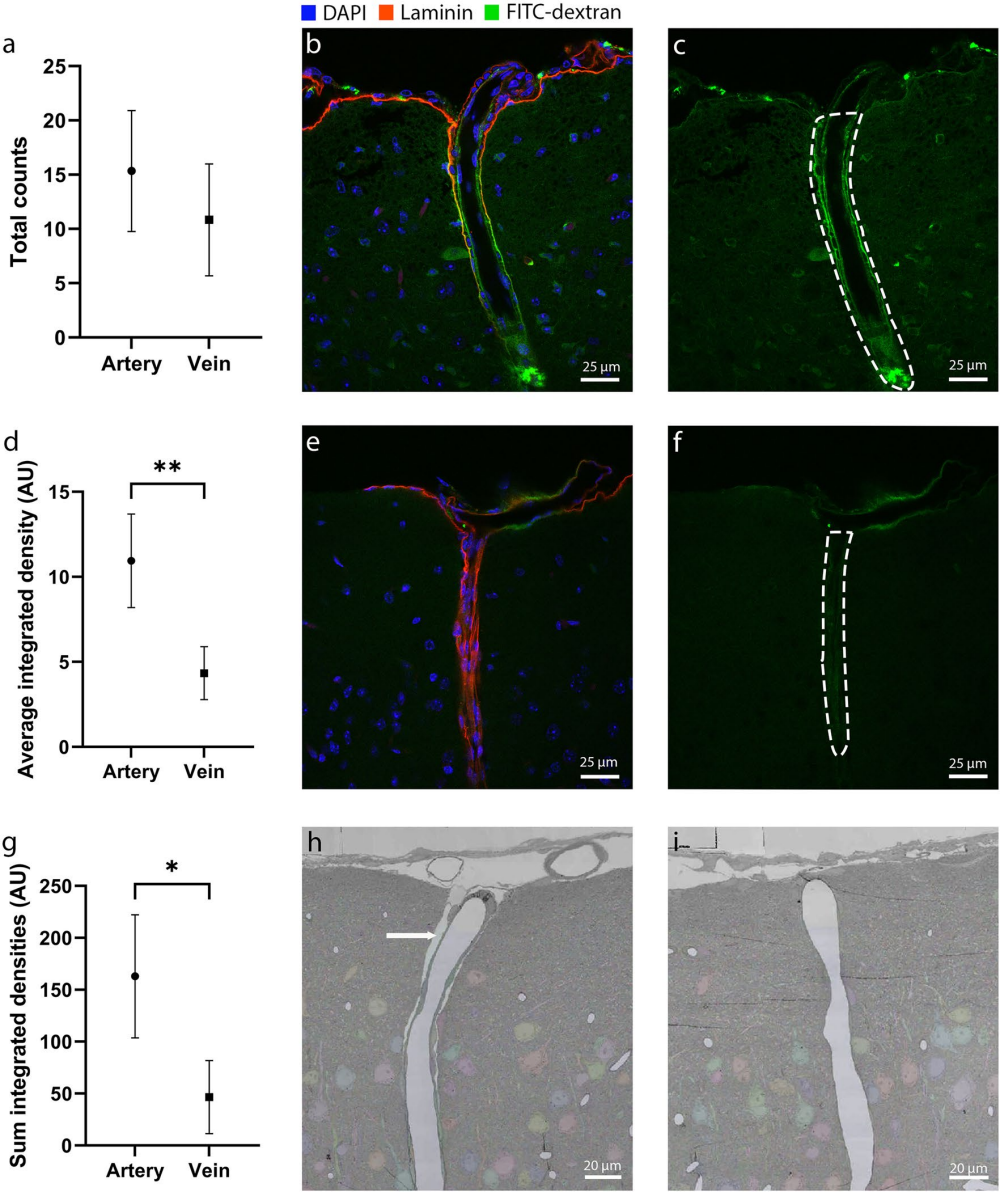
(**a**) Total number of penetrating blood vessels with CSF tracer seen in a section of one hemisphere per animal (n = 6). (**b**) Microscopic image of perivascular signal around an artery penetrating brain tissue. (**c**) FITC-dextran signal of the artery in 5b. The dashed line represents the calculated area. (**d**) Average integrated densities of CSF tracer for all animals combines per artery and vein (n = 6). (**e**) Microscopic image of perivascular signal in green around a vein the subarachnoid space and penetrating the brain tissue. (**f**) FITC-dextran signal of the vein in 5e. The dashed line represents the calculated area. Both vessels are obtained from the same animal and imaged with similar laser power and post-imaging processing. (**g**) Sum of integrated densities of the CSF tracer for all animals combined per artery and vein (n = 6). (**h**) EM database example of a penetrating artery. The perivascular space is indicated by the arrow. (**i**) Example of EM database showing a penetrating vein with no perivascular space.

Further histological analysis of the brain sections was done to quantify the FITC signal associated with penetrating arteries and veins. The data showed that although the average number of penetrating arteries with CSF tracer did not differ from the average number of veins (p = 0.303, N = 6; Fig. 5a), the penetrating arteries and veins did show differences in the amount of CSF tracer signal associated with the vessels (Figs. 5b,c,e,f). Thus, on average the penetrating arteries showed a significantly higher integrated density compared to veins (p < 0.01; Fig. 5d). Furthermore, the sum of all penetrating arteries showed a higher total amount of FITC signal (integrated density) as compared to veins (p < 0.05; Fig. 5g). Finally, the EM database showed that penetrating arteries had a clearly identifiable PVS, whereas the PVS of penetrating veins was present only superficially (Figs. 5h,i).

## Discussion

### Perivascular spaces are larger and more abundant around arteries compared to veins

This study provides the first direct comparison of the anatomical characteristics of the PVS around both pial and penetrating arteries and veins in mice. It was observed that the PVS of pial arteries is not only larger than that of veins, but it also exhibited more consistent positive tracer labeling. In this context, all arterial vessels showed labeling, whereas approximately 38% of pial veins were labeled. The size of both pial arterial and venous PVS was found to correlate with the size of the vessel, maintaining an area ratio of about 0.43 for arteries and 0.35 for veins. This ratio is lower for pial arteries compared to previous reported ratios. Raicevic et al. found a PVS-to-vessel area ratio of 1.12, but used a different approach using a fit that also includes parts of the SAS^9^. Mestre et al. reported a PVS-to-vessel area ratio of 1.4, with a large variability in this ratio^8^. It is however not entirely clear how the PVS was segmented in that study.

Labeled venous PVS were often present near the arterial PVS, but signal was also absent in many cases. It should be noted that, as our findings are based on tracer infusion experiments, the absence of tracer not necessarily implies that spaces in question do not exist. A subset of venous PVS may constitute a distinct compartment that remains inaccessible to the tracer or might be labeled at later stages. Nonetheless, given that our measurements were conducted on samples fixed one hour post-tracer infusion, this duration should suffice for the tracer to distribute throughout the entire brain clearance system^18^. Previous research also displayed more CSF tracer in the perivascular spaces around arteries compared to veins, which is in line with our findings^19,20^. For penetrating vessels, a similar dominance of PVS around arteries was observed, with strong and consistent labeling, while many penetrating veins were devoid of tracer signal. In contrast to the pial vessels, no correlation between the size of the PVS and blood vessel diameter was found for these penetrating vessels in this dataset. A limitation here is that image quality decreased with increased penetration depth, leading to more variability in the measured values.

### Meningeal sheets delineate the perivascular space in the subarachnoid space

Perivascular spaces of pial vessels were separated from the SAS by a thin meningeal sheet. In fact, this separation allowed for a clear segmentation of the PVS. This structure was seen to cover multiple arteries and veins at once when vessels ran in close proximity, connecting their PVS. However, tracer signal was present both in the PVS and the SAS. This indicates that at least for tracers up to 70 kDa, as used in the present study, this sheet does not impose a significant barrier. This observation is in line with our previous work, where we found that molecules of 200 kDa spread across arterial and venous PVS and the SAS, suggesting continuity of these structures^21^. Larger particles however, such as microspheres, appear to remain in arterial PVS in vivo^7^. The sheet we observed appeared similar to the structure referred to as SLYM in recent papers^17,22^. However, in those papers tracers were found not to cross the membrane, unless obvious damage was inflicted. The membrane that we observed in vivo was difficult to match with structures visible in the EM database. This aligns with recent findings regarding the SLYM membrane, indicating that post-mortem collapse, fixation, and processing could hinder the identification of this membrane. However, these findings have faced online criticism^22–26^, arguing that the SLYM is not a novel layer but rather the inner layer of the arachnoid that was displaced because of a large infused volume. It is possible that it is a matter of semantics, and several studies actually refer to the same membrane as either inner arachnoid, extension of the pia mater, or perivascular leptomeningeal sheet^15,17,20,22,27^. Recently, similar findings were seen in the subarachnoid space of humans, where tracer was found in distinct spaces around arteries after injection, suggesting the presence of a perivascular membrane^28^. This membrane becomes more pronounced at locations were pial vessels dive into brain tissue. Thus, in the online EM database, we did consistently observe a similar sheet extending from the pia mater, covering the PVS upon entry of the brain parenchyma. Mestre et al. recently described the linings of meningeal sheets surrounding penetrating PVS in detail^11^. Varying orientations of pia mater in the PVS were found, but these were found not to obstruct tracer influx, confirming that the penetrating PVS is essentially continuous with the subarachnoid space PVS for most solutes^11^.

As previously mentioned, the high permeability of meningeal membranes suggests a path of low resistance for fluid transfer between the PVS of arteries and veins within the SAS. Arteries and veins often intersect in the SAS without any clear anatomical barrier separating their PVS. This arrangement negates the potential for a pressure gradient between the arterial and venous PVS. Crucially, this "short circuit" scenario does not align well with theories of brain clearance that depend on interstitial flow, since bulk flow requires a pressure or concentration gradient to occur. Further work is therefore needed to unravel what determines influx of tracer into the tissue along penetrating vessels vs. tracer flux that bypasses brain tissue and directly moves from arterial to venous PVS.

### Signal associated with perivascular spaces along penetrating vessels

Histological analysis of the sections showed a clear difference in tracer signal intensity and distribution associated with penetrating arteries as compared to veins. Both the average FITC signal per vessel as well as its sum was significantly higher around arteries as compared to veins. This suggests that the role of penetrating arteries in solute exchange between the brain interstitium and CSF is larger. This may be a direct consequence of the size of the PVS along penetrating arteries. EM data revealed a distinct PVS along arteries in post-mortem samples. In contrast, a PVS surrounding veins was only evident at the tissue's entry point and was scarcely visible deeper within the tissue. In vivo and EM data showed larger periarterial spaces at the entry into brain tissue, while this diminishes as the artery further penetrates the brain tissue, which can also be seen in previous literature^29^. Collectively, these findings imply that the PVS around arteries is more effectively scaled for solute exchange compared to that around veins.

### Lack of intramural tracer in vivo

Tracer was observed adjacent to the arterial wall in vivo, but clearly visible within the extracellular matrix around smooth muscle cells in the arterial wall of ex vivo brain sections. This observation likely resulted from the post-mortem processing of the brain, as well as the collapse of the PVS following death^30^. Mestre et al. showed that perfusion fixation caused retrograde flow of the CSF tracer which led to a collapse of the PVS, resulting in the CSF tracer entering the arterial wall^8^. While other studies have indicated a drainage pathway within the arterial wall^31^, a direct comparison of our in vivo and histological data suggests that the intramural signal is likely an artifact of post-mortem brain tissue preparation and analysis^30^.

### Limitations

Our study detailed the dimensions of PVS, the number of labeled vessels, the amount of tracer signal associated with blood vessels, and the precise location of tracer with respect to the vessel wall. Yet, it represented merely a single moment in time, specifically one hour post-tracer infusion. When observing tracer distribution after CSF infusion over time by two-photon imaging, tracer was present in the veins after 45 min, suggesting that the one hour time point is suitable for investigating tracer distribution in the PVS^19^. Nevertheless, it remains plausible that the parameters we quantified could vary over time and depend on physiological conditions. Time-dependent monitoring of perivascular tracer dispersion in perivenous and periarterial spaces could provide additional information on brain clearance dynamics.

## Conclusions

In summary, this study addressed the structural characteristics and prevalence of the PVS associated with the brain’s arteries and veins. Given their larger dimensions and the greater tracer association, periarterial spaces seem to play a more significant role in the exchange of solutes between the CSF and brain parenchyma compared to perivenous spaces. Future research is essential to delineate the flow dynamics within the perivenous spaces, enhancing our comprehension of the brain's waste clearance system.

### Supplementary Information

Supplementary Video 1.

## Data Availability

The datasets used and/or analysed during the current study are available from the corresponding author on reasonable request.
